# CROSS-NATIONAL COMPARISON OF DISABILITY IN LATIN AMERICAN OLDER ADULTS

**DOI:** 10.1093/geroni/igae098.0311

**Published:** 2024-12-31

**Authors:** Margarita Maria Osuna, Jennifer Ailshire

**Affiliations:** University of Southern California Leonard Davis School of Gerontology, Los Angeles, California, United States; University of Southern California, Los Angeles, California, United States

## Abstract

Latin America’s population is aging rapidly. Little is known about disability prevalence among older adults in these countries. We estimate and compare disability prevalence from nationally representative surveys of older adults from Mexico (2015 Mexican Health and Aging Study, MHAS, N=6,699), Brazil (2015 Brazilian Longitudinal Study of Aging, ELSI, N=5,367), Colombia (2015 National Survey of Health and Wellbeing, SABE-Col, N=23,598), and Costa Rica (2005 Costa Rican Longevity and Healthy Aging, CRELES, N=2,607). Disabilities were defined using three harmonized measures: any limitations on (i) instrumental activities of daily living (IADL), (ii) activities of daily living (ADL), and (iii) mobility. Age-adjusted prevalence estimates were calculated by gender for each country, using WHO 2000-2025 Standard Population age categories. Findings reveal significant differences in disability prevalence across measures and countries. Prevalence of IADL disability in men ranged from 18% (Costa Rica) to 31% (Brazil). For women, IADL disability ranged from 24% (Colombia) to 30% (Brazil). ADL disability for men ranged from 10% (Brazil) to 20% (Mexico). Whereas women’s prevalence range was 12% (Costa Rica) to 23% (Mexico), mobility disability exhibited the highest prevalence and had the largest disparities. Prevalence in men ranged from 27% (Colombia) to 78% (Mexico). Women’s disability prevalence ranged from 43% (Colombia to 83% in Mexico. Findings highlight the heterogeneity of health inequalities and raise policy implications. Aging and healthcare policies should focus on supporting older adults, with stronger social protections and home improvements to support disabled older adults. Our study provides insights on disability patterns in Latin America.

